# HIV Prevalence in a Gold Mining Camp in the Amazon Region, Guyana

**DOI:** 10.3201/eid0803.010261

**Published:** 2002-03

**Authors:** Carol J. Palmer, Lloyd Validum, Bernard Loeffke, Harold E. Laubach, Chris Mitchell, Rudy Cummings, Raul R. Cuadrado

**Affiliations:** * Nova Southeastern University, Ft. Lauderdale, Florida, USA; † BioQuest, Miami, Florida, USA; ‡ Woodlands Hospital, Georgetown, Guyana; § Ministry of Health, Georgetown, Guyana

**Keywords:** HIV, Amazon, Guyana, South America, indigenous peoples, gold mining, malaria, coinfection

## Abstract

The prevalence of HIV infection among men in a gold mining camp in the Amazon region of Guyana was 6.5%. This high percentage of HIV infection provides a reservoir for the virus in this region, warranting immediate public health intervention to curb its spread. As malaria is endemic in the Amazon Basin (>30,000 cases/year), the impact of coinfection may be substantial.

In Guyana and other South American countries containing large tracts of Amazon jungle, few studies have investigated the prevalence of HIV infection in isolated communities (*1*). Geographic isolation would lead to low infection rates because of lack of exposure to the disease. In addition, prevalence data on HIV can be negligible even in urban areas. For example, the only reported HIV studies in the Guyanese population have focused on HIV prevalence in commercial sex workers in the capital city of Georgetown (*2*). No studies have reported the prevalence of HIV in Guyanese men or in the interior Amazon region of the country. We evaluated a group of men living and working in the Amazon region of Guyana to determine the prevalence of HIV infection.

This study was conducted after we obtained Institutional Review Board approvals as well as permission from the Guyanese Ministry of Health and the director of a local Guyanese gold mining camp. Informed consent was obtained from each participant. Typically, men live in gold mining camps for periods of 6 to 8 weeks, working 12-hour days, 7 days per week. At the end of a 6- to 8-week shift, the men rotate out of camp to their homes on the coast for 2 weeks of rest. Mining gold in the Amazon region requires considerable manual labor and long hours working in a hot, humid jungle environment. Salaries paid to gold miners, however, are much better than those of typical jobs in the city. Thus, jobs in the mining industry are attractive, and many men leave their families and work as miners in the jungle for a few years to provide a better standard of living for their families.

The mining camp in this study was approximately 400 km inland from Georgetown, the capital of Guyana, in the heart of the Amazon region of the country. It was typical of many of the mining camps in the jungle (Figure). Men sleep in rows of 20 to 40 hammocks strung underneath a large tarp-like covering. The tarp coverings are not enclosed, but the men usually sleep under mosquito netting, as malaria infection is a constant problem. Pit latrines are available in the camp as are rainwater shower stalls. Water is obtained from a nearby stream, and a generator provides light in the camp in the evenings. The facility is fenced in and heavily guarded by armed patrols, as gold is stored in the camp from daily operations. The camp is a living facility only as all mining occurs outside the camp itself.

**Figure:**
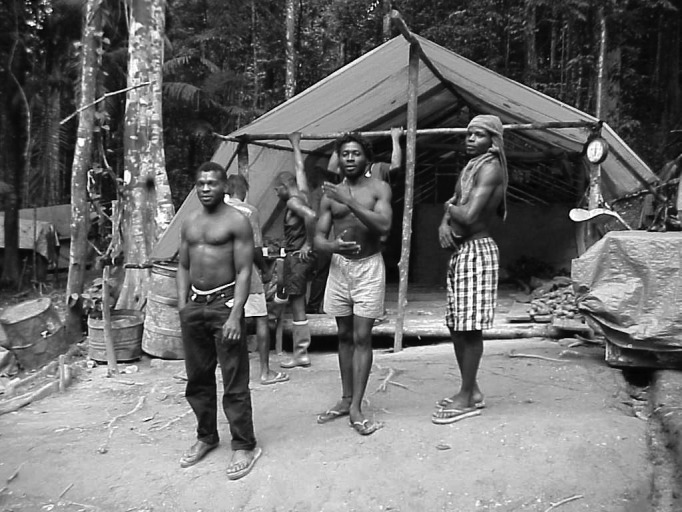
Typical living quarter for miners in the jungle, Amazon Basin, Guyana.

We enrolled almost the entire workforce of the mining camp (n = 216) for participation in this study. Only four declined the free HIV test and were excluded from the analysis. All 216 subjects were Guyanese men (age range 18-35 years). Pre- and post-HIV counseling was completed, and informed consent was obtained from all participants. Seven milliliters of venous blood was obtained from each participant after precounseling was completed. Onsite screening for HIV was completed, and serum was stored on ice and transported for confirmatory testing in a laboratory. Onsite HIV testing was by the Determine (Dainabot, Tokyo, Japan) rapid immunochromatographic test for the qualitative detection of HIV-1/2; in previous field work this test yielded 100% sensitivity and specificity (*3*). The test required 50 :L of serum, with results available for visual interpretation within 15 to 60 minutes. However, most of the positive samples produced a clearly visible red line within 10 minutes. HIV testing by enzyme-linked immunosorbent assay (ELISA) with Western blot confirmation (Abbott, Abbott Park, IL) was completed on all sera on our return from the jungle. Participants were not given results until confirmatory testing was completed. All participants were notified that results were available by a letter from the collaborating local physician, and all were offered personal counseling when they visited him for their results.

Fourteen (6.5%) of the 216 men were found to have HIV infection by results of both the onsite rapid strip test and subsequent ELISA and Western blot tests. Results obtained with the rapid test performed onsite had 100% agreement with those of the laboratory tests performed after our return.

Results of this small HIV screening study, indicating that 6.5% of men living in this remote camp were HIV positive, suggest enormous potential for further transmission of HIV in Guyana, in both jungle and urban environments. Migration of city dwellers into the Amazon jungle region may increase the risk of transmission of HIV to indigenous people. Conversely, gold miners can become infected with HIV during contact with commercial sex workers in small towns near the mining areas. HIV can then be further transmitted to the miner’s spouse and unborn children on his return to the city. Whether the miners contracted the infection while living in the jungle or whether they entered the region already infected is unclear; however, the high percentage of HIV infection in this population provides a reservoir for the disease in this region and warrants immediate public health intervention to curb its spread.

Intervention is warranted to increase public awareness of HIV in underserved remote jungle and urban regions in Guyana. Rapid HIV screening tests, which can be completed without equipment or ancillary supplies, may provide an important tool for rapid screening and providing immediate feedback to patients. Initial counseling on risk-reducing behavior can be initiated onsite to provide an immediate intervention strategy to prevent the spread of the disease while follow-up testing with a confirmatory HIV test is completed.

Given the high numbers of malaria cases in the Guyana Amazon region, combined with this new evidence of potentially escalating HIV rates, studies are warranted to measure the impact of HIV/malaria coinfection. Reports showing an average of >30,000 cases of malaria per year over the past decade clearly designate this region as having a high rate of endemic malaria (*4*). Since T-cell and B-cell function, thought to provide a defense against malaria, are both adversely affected at the early stages of HIV infection and continue to deteriorate, this may contribute to higher rates of malaria mortality or more severe malaria symptoms, as the infected person’s impaired immune system is less effective against the invading parasites. Conversely, malaria could exacerbate HIV infection (*5*), since the already compromised immune system may be overwhelmed by the multiple infection. Thus, HIV/malaria coinfection may contribute to increased rates of illness and death in the Amazon region. Recent studies on HIV/AIDS and malaria in Africa suggest that coinfection with these two diseases has become a concern in Africa (*6*,*7*). This problem also merits attention in the Americas so that further research, planning, and interventions will be focused in this region.
